# *Trans*-cinnamaldehyde nanoemulsion reduces *Salmonella* Enteritidis biofilm on steel and plastic surfaces and downregulates expression of biofilm associated genes

**DOI:** 10.1016/j.psj.2025.105086

**Published:** 2025-03-22

**Authors:** Trushenkumar Shah, Chen Zhu, Chetna Shah, Indu Upadhyaya, Abhinav Upadhyay

**Affiliations:** a Department of Animal Science, University of Connecticut, Storrs, Connecticut, USA; b Department of Extension, University of Connecticut, Storrs, Connecticut, USA

**Keywords:** *Salmonella* Enteritidis, Biofilm, Poultry, *Trans*-cinnamaldehyde nanoemulsion, Natural sanitizer

## Abstract

*Salmonella* Enteritidis is a major poultry-associated foodborne pathogen that can form sanitizer-tolerant biofilms on various surfaces. The biofilm-forming capability of *S*. Enteritidis facilitates its survival on farm and food processing equipment. Conventional sanitization methods are not completely effective in killing *S*. Enteritidis biofilms. This study investigated the efficacy of a Generally Recognized as Safe phytochemical Trans-cinnamaldehyde (TC), and in its nanoemulsion form (TCNE), for inhibiting *S*. Enteritidis biofilm formation and inactivating mature biofilms developed on polystyrene and stainless-steel surfaces. Moreover, the effect of TC on *Salmonella* genes critical for biofilm formation was studied.

TCNE was prepared using a high energy sonication method with Tween 80. For biofilm inhibition assay, *S*. Enteritidis was allowed to form biofilms either in the presence or absence of sub-inhibitory concentration (SIC; 0.01 %) of TCNE at 25°C and the biofilm formed was quantified at 24-h intervals for 48 h. For the inactivation assay, *S*. Enteritidis biofilms developed at 25°C for 48 h were exposed to TCNE (0.5, 1 %) for 1, 5, and 15 min, and surviving *S*. Enteritidis in the biofilm were enumerated. SIC of TCNE inhibited *S*. Enteritidis biofilm by 45 % on polystyrene and 75 % on steel surface after 48 h at 25°C compared to control (*P* < 0.05). All TCNE treatments rapidly inactivated *S*. Enteritidis mature biofilm on polystyrene and steel surfaces (*P* < 0.05). The lower concentration of TCNE (0.5 %) reduced *S*. Enteritidis counts by 1.5 log CFU/ml as early as 1 min of exposure on both polystyrene and stainless-steel surfaces. After 15 min of exposure, TCNE at concentration of 0.5 or 1 % reduced *S*. Enteritidis count significantly by 4.5 log CFU or 6 log CFU/ml on polystyrene or stainless-steel surfaces. TC downregulated the expression of *S*. Enteritidis genes (*hilA, hilC, flhD, csgA, csgD, sdiA)* responsible for biofilm formation (*P* < 0.05). Results suggest that TCNE has potential as a natural disinfectant for controlling *S.* Enteritidis biofilms on common farm and food processing surfaces, such as plastic and steel.

## Introduction

In the Unites States, non-typhoidal *Salmonella* causes ∼ 1.35 million infections, 26,500 hospitalizations, and 420 deaths every year (CDC, 2022). More than 2,600 *Salmonella* serotypes have been identified (Grimont and Weill, 2007); however, *Salmonella enterica* subspecies enterica serovar Enteritidis is considered to be one of the major zoonotic non-typhoidal *Salmonella* serovars associated with foodborne infections (Ferrari et al., 2019). Poultry meats and eggs are considered the major food vehicles for the transmission of *S.* Enteritidis (Karabasanavar et al., 2020; EFSA and ECDC, 2021; Guillier et al., 2021) and several outbreaks have been reported in the past decades (EFSA and ECDC, 2023). Thus, control measures are implemented in poultry farms as well as in processing environments to reduce *Salmonella* transmission.

Bacteria, especially foodborne and nosocomial origins, have developed a variety of strategies to adapt and survive in stressful environments. The formation of biofilms is one such strategy which facilitates survival and persistence of microbes in various environmental conditions (Hall-Stoodley et al., 2004; De la Fuente-Nunez et al., 2013). Biofilms are associations of numerous bacterial cells, embedded in a self-produced extracellular polymeric matrix and attached to a surface (Hall-Stoodley et al., 2004; Bjarnsholt, 2013). *Salmonella* biofilm formation involves several distinct stages, which are crucial for its persistence and pathogenicity. *Salmonella* cells adhere to surfaces using fimbriae and flagella (Horstmann et al., 2020). This reversible attachment is influenced by environmental conditions and surface properties. In the subsequent stage, cells produce extracellular polymeric substances (EPS), strengthening their attachment and beginning the formation of microcolonies. This stage marks the transition from reversible to irreversible adherence​ (Beshiru et al., 2018, Shatila et al., 2021)​​. Bacterial dispersal into the environment marks the final stage of the biofilm life cycle and contributes to biological dispersal, bacterial survival, and disease transmission (Abdullahi et al., 2016). Biofilms increase virulence and persistence of the underlying pathogen in food processing environments (Steenackers et al., 2012; Warrier et al., 2021). In addition, recent research has shown that biofilms can also act as hotspots for horizontal gene transfer of antibiotic resistance genes (Sharma et al., 2019; Flores-Vargas et al., 2021; Michaelis and Grohmann, 2023).

*Salmonella* is known to form biofilm on various surfaces such as steel (Paz-Méndez et al., 2017), plastic (Stepanović et al., 2004), rubber (Carrascosa et al., 2021), and even eggshells (Lee et al., 2020). The biofilm forming capability of *Salmonella* increases tolerance to antimicrobials, disinfectants, and other environmental stresses (Bridier et al., 2011; Esbelin et al., 2018; Cadena et al., 2019; Tassinari et al., 2019). This allows the survival of *Salmonella* for long periods of time in farm or processing environments (Steenackers et al., 2012; O'Leary et al., 2015; Moraes et al., 2018). Recent reports have also identified a relationship between *in vivo* pathogenicity of *Salmonella* and their biofilm formation capability (Borges et al., 2018). Moreover, literature suggests that *Salmonella* biofilm formation at farm or processing plants lead to contamination of poultry or poultry products (Chitlapilly Dass and Wang, 2022; Wang et al., 2022).

Currently, poultry industries use chemical-based interventions for controlling *Salmonella* biofilms in farm and processing environments. These include halogens such as hypochlorite, peroxygens like hydrogen peroxide, peracetic acid (PAA), and quaternary ammonium compounds (Chmielewski and Frank, 2003, Kabir et al., 2021, Obe et al., 2021; Pablos et al., 2022). Chlorine is another commonly used antimicrobial agent, being administrated at up to 50 ppm in the wash and chiller steps of poultry processing (USDA FSIS, 2024). However, it has been shown to have reduced efficacy in the presence of organic matter (Van Houdt and Michiels, 2010). Therefore, there is a need to develop novel disinfection strategies to control *S.* Enteritidis biofilm in farm and processing environments.

The use of phytochemicals for sanitation, cooking and medicinal purposes dates back thousands of years, spanning across various cultures and civilizations. The majority of phytochemicals are secondary metabolites produced as a defense mechanism to protect plants from pathogenic microorganisms (Borges et al., 2016). Several phytochemicals have been evaluated for their antimicrobial effect against foodborne pathogens (Burt, 2004; Holley and Patel, 2005; Upadhyay et al., 2014). *Trans*-cinnamaldehyde (TC) is one such phytochemical obtained from bark extract of cinnamon (*Cinnamomum zeylandicum*) and has been classified as Generally Recognized as Safe compound (approval TC-21CFR182.60) by the United States Food and Drug Administration. TC exerts its antimicrobial effect against both Gram-positive and Gram-negative microorganisms (Kollanoor-Johny et al., 2008; Jo et al., 2015; Upadhyaya et al., 2015; Upadhyay and Venkitanarayanan, 2016). Previous studies have demonstrated the antibiofilm efficacy of TC against pathogens such as *Listeria monocytogenes* (Upadhyay et al., 2013), *Campylobacter jejuni* (Wagle et al., 2019a), *Escherichia coli* (Olszewska et al., 2020), *Salmonella* Typhimurium (Yin et al., 2022), *Staphylococcus aureus* and *S.* Enteritidis (Zhang et al., 2014; Cabarkapa et al., 2019; Somrani et al., 2022).

While TC has been investigated as an antimicrobial to eradicate bacterial biofilms on various surfaces at the pre- and postharvest level, the low solubility of TC in water presents several challenges in its application as a water-based disinfectant (Doyle and Stephens, 2019; FAO, 2023; PubChem, 2024). Therefore, to overcome this challenge and improve the dispersion and antimicrobial efficacy of TC in water, TC nanoemulsion (TCNE) was prepared using high energy sonication with Tween 80 as emulsifier. Thereafter, the efficacy of TCNE treatments in reducing *S.* Enteritidis biofilm on polystyrene and stainless-steel surface was investigated. In addition, the effect of TC treatments on biofilm architecture and genes critical for *Salmonella* biofilm formation were determined.

## Materials and methods

### S. Enteritidis strain and culture conditions

*S.* Enteritidis strain, SE 21 (isolated from chicken intestine) was used in this study. The organism was streaked from the glycerol stock on XLD agar plates, followed by incubation at 37℃ for 48 h. Individual colonies were selected and cultured in 10 ml of tryptic soy broth (Fisher Scientific Co LLC, Hanover Park, IL) at 37℃ for 24 h. For inoculum preparation, the individual overnight culture was centrifuged at 7000 rpm for 15 min at 25℃. The bacterial pellet was washed three times and resuspended in 10 ml of 1X phosphate buffered saline (PBS, pH 7.0). The concentration of the bacterial population in the resuspended culture was confirmed by spectroscopy (OD-600 nm) and plating on the Xylose Lysine Deoxycholate **(**XLD) agar followed by incubation at 37℃ for 24-48 h. The resuspended culture was diluted appropriately as per the need of the experiments.

### Preparation and characterization of trans-cinnamaldehyde nanoemulsion

An oil-in-water nanoemulsion of TC was prepared using sonication, a high energy method. Trans-cinnamaldehyde (99 %, catalogue no. AC110350010, Fisher Scientific, Waltham, MA) was mixed with Tween 80 (catalog no. 28329, Fisher Scientific), a synthetic non-ionic surfactant. The TCNE was prepared as 5 % stock solution as per previously published protocol (Bhargava et al., 2015). Hydrophobic oil and surfactant were mixed at a 2:1 mass ratio for 30 min at constant speed (∼400 rpm). Under continuous stirring, deionized (DI) water was added dropwise and stirred for 30 min at 23°C. The solution was sonicated using high speed homogenizer (QSonica 700, QSonica L.L.C, Newtown, CT) for 20 min with 5 s On and 3 s Off cycle and an amplitude of 75 Watts. The prepared nanoemulsion was stored at 4°C for 2 months. The stability of the nanoemulsion was measured by characterizing the size, polydispersity index (PDI), and zeta potential on month 0, 1, and 2 using Nano Zetasizer.

### Determination of MBC, MIC and SIC of TC or TCNE

The minimum bactericidal concentration (MBC), minimum inhibitory concentration (MIC) and sub-inhibitory concentration (SIC) of TC or TCNE were determined as previously described (Upadhyay et al., 2013). One ml of TSB containing ∼6.0 log CFU/ml of *S.* Enteritidis was added to sterile 24-well polystyrene tissue culture plates (Costar, Corning Incorporated, Corning, NY) followed by the addition of 1 ml of TSB containing various doses of TC or TCNE (Sigma–Aldrich) ranging from 0.008 to 0.1 %. The plates were incubated at 37°C for 24 h, and bacterial growth was determined by culturing on duplicate Tryptic Soy Agar (TSA) and XLD agar plates. The lowest concentration of TC or TCNE that reduced *S.* Enteritidis population in TSB by ∼5.0 log CFU/ml after incubation at 37°C for 24 h was taken as the MBC (CLSI, 2019). The lowest concentration of TC or TCNE that inhibited the growth of *S.* Enteritidis after incubation at 37°C for 24 h was taken as the MIC. The two highest concentrations of TC or TCNE below its MIC that did not inhibit bacterial growth after 24 h of incubation as compared to control samples were selected as its SICs for this study.

### Biofilm inhibition assay on polystyrene plates

The ability of TC or TCNE in inhibiting *S.* Enteritidis biofilm formation on polystyrene plates was determined according to a previously published method (Reeser et al., 2007; Lu et al., 2012). Briefly, 1 ml of *S.* Enteritidis culture (∼6.0 log CFU) was added to each well of a sterile 24-well polystyrene plate, followed by addition of SIC of TC or TCNE. The plates were incubated at 25°C for 48 h and biofilm formation was determined at 24 h intervals. At each time point, the spent medium was removed. The well containing the biofilm was gently washed three times with 1X PBS and 1 ml Dey-Engley (DE) neutralizing broth was added (Difco Laboratories, Sparks, MD, United States). The biofilm was removed from the polystyrene plate's surface using a micro-scraper. The DE broth suspension containing the biofilm cells was serially diluted and surface plated on XLD agar plates followed by incubation at 37℃ for 24 - 48 h for pathogen enumeration (Upadhyay et al., 2013).

### Biofilm inactivation assay on polystyrene plates

The inactivation of mature *S.* Enteritidis biofilms by TC or TCNE was determined as described previously by Kim et al. (2017) with slight modification. Briefly, 1 ml culture of *S.* Enteritidis (∼6.0 log CFU) was allowed to form biofilm in 24-well polystyrene plate at 25°C for 48 h. After mature biofilm was formed, the inactivation was carried out with 1 ml treatment solution of TC or TCNE at concertation of 0.5 and 1 % for 1, 5, or 15 min. The treatment solution was removed, and 1 ml of DE broth was added to the polystyrene plate wells. The surviving *S.* Enteritidis in the biofilms were removed using a micro-scraper and DE broth suspension was serially diluted, and surface plated on XLD agar plates followed by incubation at 37℃ for 24 - 48 h for pathogen enumeration (Upadhyay et al., 2013).

### Preparation of stainless-steel coupons

Stainless steel coupons (type 304; diameter 1 cm) with no. 4 finish were prepared, as previously described method (Jeong and Frank, 1994). Briefly, steel coupons were cleaned with acetone followed by washing in distilled water and soaking in 100 % ethanol. Finally, steel coupons were rinsed with distilled water, air dried and autoclaved at 121°C for 15 min.

### Biofilm inhibition assay on stainless steel coupons

The inhibition of *S.* Enteritidis biofilm formation by the TC or TCNE on stainless steel coupons was determined by method described by Upadhyay et al. (2013). Briefly, sterile stainless-steel coupons were individually placed in each well of a 24- well cell culture plate (Corning™ Costar™ flat bottom cell culture plates) containing 1 ml of TSB with or without (control) SIC of TC or TCNE. *S.* Enteritidis (∼6.0 log CFU/ml; 1 ml in TSB) was added to each well and incubated at 25℃ for 48 h. Stainless coupons were collected after 24 and 48 h and washed with 1 ml of sterile deionized water (DI) with gentle agitation for 5 s and transferred to sterile centrifuge tube containing 1 ml of DE broth with sterile glass beads. The tubes were vortexed for 1 min to detach the biofilm containing *S.* Enteritidis from the coupons. The DE broth suspension was processed for pathogen enumeration as described above.

### Biofilm inactivation assay on stainless steel coupons

For the inactivation of mature biofilm on steel coupons, mature *S.* Enteritidis biofilms were developed on sterile coupons placed in 24-well polystyrene plates containing *S.* Enteritidis (∼6.0 log CFU) at 25°C for 48 h. The coupons were rinsed in 1 ml of sterile DI water three times with gentle agitation for 5 s, transferred to a sterile 24 well polystyrene plates containing TC or TCNE at 0.5 and 1 %, and incubated at 25℃ for 1, 5, or 15 min. After treatment, the coupons were transferred to centrifuge tube containing 1 ml of DE broth with sterile glass beads. The tubes were vortexed for 1 min to detach biofilm containing *S.* Enteritidis from the coupons. The DE broth suspension was serially diluted and surface plated (0.1 ml, in duplicate) on XLD agar plates, and incubated at 37℃ for 24-48 h (Upadhyay et al., 2013).

### Bacterial viability assay

To study the effect of treatments on biofilm architecture, bacterial viability assay was performed using a Leica true confocal scanner SP2 microscope with a water immersion lens (Balasubramanian et al., 2023). Biofilms were formed at 25℃ on a Lab-Tech four-chamber no. 1 borosilicate glass coverslip system (Lab-tek, Nalge Nunc International, Rochester, NY) for 48 h in TSB. Thereafter, the biofilms developed on coverslips were exposed to 1 % TC or TCNE and live and dead bacteria in the biofilm were imaged at 63x magnification after staining with 0.0025 mM SYTO (Molecular Probes, Oregon) and 0.005 mM propidium iodide (PI; Molecular probes).

### Gene expression analysis of S. Enteritidis exposed to trans-cinnamaldehyde

The effect of TC on the expression of genes critical for *Salmonella* biofilm formation and surface attachment was determined using real-time quantitative PCR (RT-qPCR) (Upadhyay et al., 2017). Briefly, *S.* Enteritidis (∼6.0 log CFU/mL) was incubated in the presence or absence of SICs of TC at 25°C for 24 h. The total RNA was extracted using RNA mini kit (Invitrogen, Carlsbad, CA, United States) and complementary DNAs were prepared using iScript cDNA synthesis kit (Bio-Rad Laboratories, Inc., CA, United States). The primers (Table 1) were obtained from Integrated DNA Technologies, Inc. (Coralville, IA, United States). The amplified products were detected by using SYBR Green reagents (Bio-Rad Laboratories, Inc.). The 16 s rRNA gene was used as the endogenous control and comparative critical threshold (ΔΔCt) method was employed to analyze relative expressions of candidate genes on Quant Studio 3 real-time PCR system (Applied Biosystems, Thermo Fisher Scientific).

**Table 1:** Primers used for gene expression analysis using real-time quantitative PCR.

Gene	Forward primer	Reverse primer
sipA	TCTGCTTTTTTCCCACCATCA	AGATAAACTGCCTGACCCTAAAATTC
sipB	GCCACTGCTGAATCTGATCCA	CGAGGCGCTTGCTGATTT
sipC	ATGTCTAGA CCCTAAATAAAGTGGCG	ATTAG ATCTCTCCCTTTATTTGGCAG
sipD	ATTCCGCTTCTCCTCATCCG	ACCGCGATGTTCTGTGGTAG
sopB	GTGCTGCAATAAGTTCGATAA GATTT	ACCGGCCAGCAACAAAAC
invA	ACAGTGCTCGTTTACGACCTGAAT	AGACGACTGGTACTGATCGATAAT
flhD	CGTTTGATCGTCCAGGACAA	TGTTTGCCATCTCTTCGTTGAT
hilA	TTGCTGACTCAATGCGTTAACA	CATTCTGCCAGCGCACAGTA
hilC	CCAGTTTTCGCTTCAGACTTGA	CACCCGCAAATGGTCACA
hilD	CAACGACTTGGCGCTCTCTAT	TCTCTGTGGGTACCGCCATT
csgA	TTACCATGAAACTTTTAAAAGTGGC	TTAATACTGGTTAGCCGTGGCGTTGTT
csgD	GCCTCATATTAACGGCGTGT	TCGCGATGAGTGAGTAATGC
sdiA	AATATCGCTTCGTACCAC	GTAGGTAAACGAGGAGCAG
rpoS	GAATCTGACGAACACGCTCA	CCACGCAAGATGACGATATG
16 s rRNA	CGTGTTGTGAAATGTTGGGTTAA	CCGCTGGCAACAAAGGATAA

### Statistical analysis

A completely randomized design was used with duplicate samples and the study was repeated three times. The data for each treatment and control were pooled from three independent trials before analysis. Bacterial counts were logarithmically transformed to maintain the homogeneity of variance for the inactivation assay (Byrd et al., 2001). The data of inhibition and inactivation assays were analyzed by least-square means analysis at *P* < 0.05 for statistical difference. The gene expression data were analyzed by Student's t-test. All analysis was performed on graph pad version 9.5 software.

## Results

### Preparation and characterization of trans-cinnamaldehyde nanoemulsion

The average particle size, PDI, and zeta potential of TCNE are presented in Table 2. The average particle size, PDI, and zeta potential of freshly prepared TCNE on day 0 were 112.8±1.14, 0.25±0.003, and -4.53±0.35, respectively. TCNE stored at 4°C maintained its particle size of ∼123.0 ± 2.46 nm and zeta potential of ∼ −5.64 ± 0.27 mV till 2 months of refrigerated storage (*P* > 0.05). However, a slight increase in PDI (∼0.05) was observed at 2 months of storage (*P* < 0.05).

**Table 2:** Effect of storage at 4°C on droplet size, poly dispersity index, and zetapotential of Trans-cinnamaldehyde nanoemulsion. Values are expressed as mean ± SE. Superscripts with different letters represent significant changes in size, PDI or zeta potential during storage.

Physicochemical properties of TCNE during refrigerated storage			
Months	Size (nm)	PDI	Zetapotential (mV)
0	112.8±1.14^a^	0.25±0.003^a^	-4.53±0.35^a^
1	121.3±2.89^a^	0.28±0.004^b^	-5.53±0.33^a^
2	123.0±2.46^a^	0.30±0.004^c^	-5.64±0.27^a^

### Sub-inhibitory concentrations and minimum bactericidal concentrations of TCNE against S. Enteritidis

TC or TCNE at 0.01 % (v/v) was the highest concentration that did not reduce the growth of *S*. Enteritidis as compared to control and was selected as the SIC for the study. The MIC of TC and TCNE was estimated to be 0.03 %. Similarly, the lowest concentration of TC and TCNE that reduced *S*. Enteritidis counts by 5 log CFU/ml was 0.06 % and this concentration was selected as the MBC for the study.

### Effect of sub-inhibitory concentrations of TC and TCNE on S. Enteritidis biofilm formation on polystyrene and stainless steel surface at 25°C

**Fig. 1:**
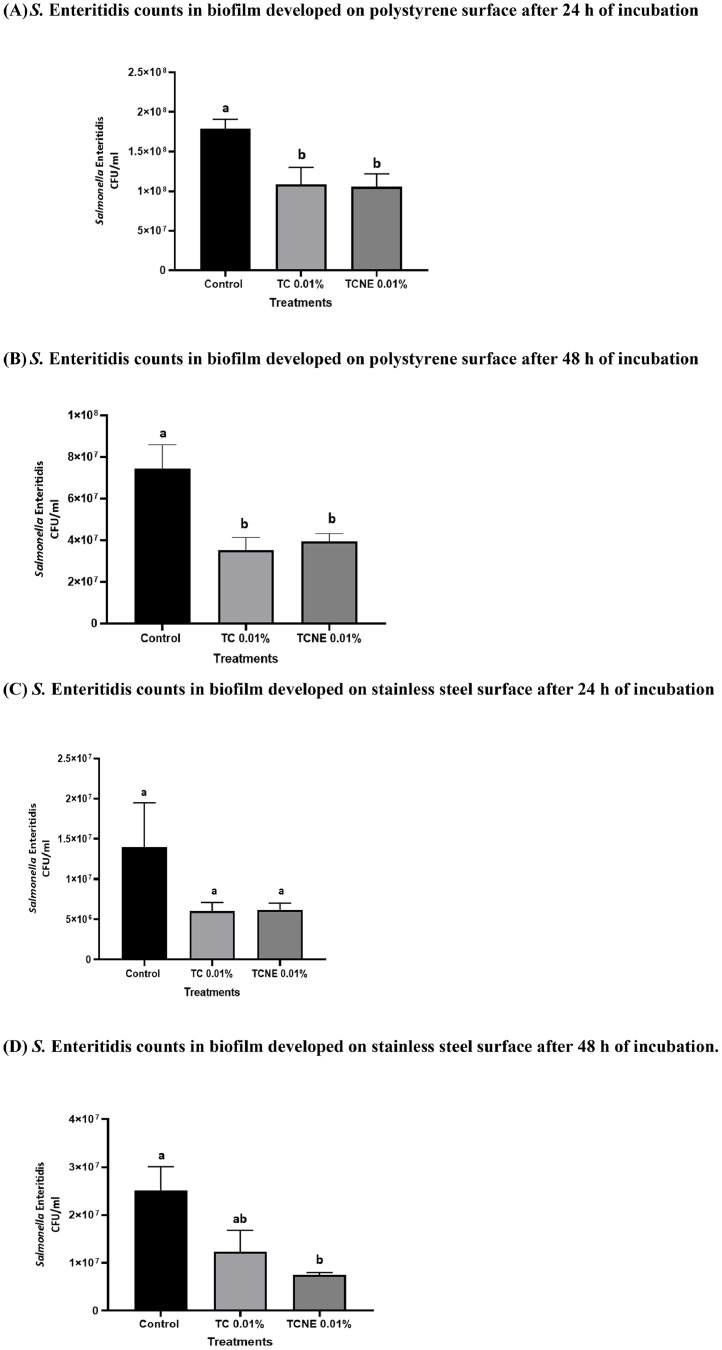
Effect of Sub-Inhibitory Concentrations of TC and TCNE on *S*. Enteritidis Biofilm Formation on Polystyrene (A&B) and Stainless Steel Surface (C&D) at 25°C. Error bars represent SEM (n = 6). *S.* Enteritidis counts in the biofilm were enumerated at 24 and 48 h.

The effect of TC and TCNE on *S*. Enteritidis biofilm formation is presented in Fig. 1. The presence of 0.01 % TC and TCNE reduced *S*. Enteritidis load in the developing biofilm by 45 % (0.3 log CFU/ml) as early as 24 h of treatment (Fig.1A). After 48 h, TC and TCNE maintained antibiofilm effect and a reduction of ∼ 45 % (0.3 log CFU/ml) was observed in *S*. Enteritidis load on polystyrene surface (Fig.1B). On steel surface, TC treatment was not effective in reducing *S*. Enteritidis biofilm formation (*P* > 0.05; Fig. 1C). However, TCNE treatment reduction biofilm formation by ∼75 % (0.5 log CFU/ml) after 48 h of incubation (*P* < 0.05; Fig. 1D).

### Efficacy of TC and TCNE in inactivating mature S. Enteritidis biofilm developed on polystyrene surface at 25°C

**Fig. 2:**
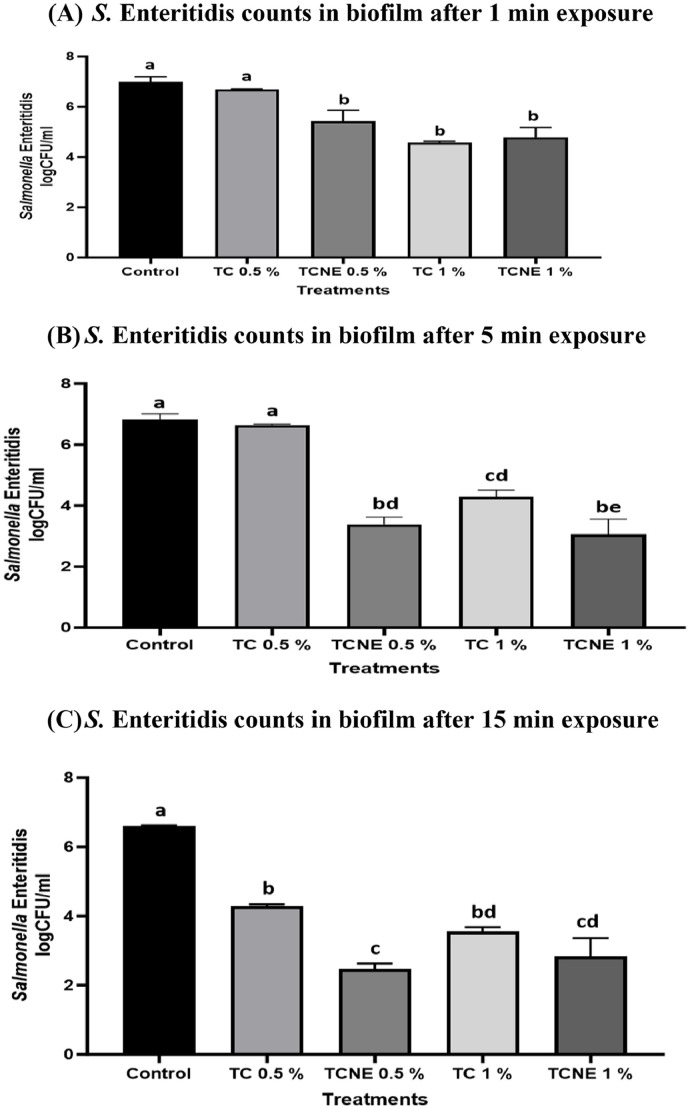
Efficacy of TC and TCNE in Inactivating Mature *S*. Enteritidis Biofilm Developed on Polystyrene Surface at 25°C (n = 6).

Efficacy of TC and TCNE in inactivating mature *S*. Enteritidis biofilm developed on polystyrene surface is presented in Fig. 2. In case of baseline (*S.* Enteritidis biofilm developed at 25°C for 48 h and not subjected to any treatment), ∼ 7 log CFU/ml of pathogen load was recovered (data not shown). Contact with DI water for 1, 5, or 15 min did not reduce pathogen load in biofilm as compared to baseline (*P* > 0.05). Exposure of biofilm to 0.5 % TC for 1 min did not reduce pathogen load in the biofilm as compared to control. However, 0.5 % TCNE reduced *S.* Enteritidis counts significantly by ∼1.5 log CFU/ml as early as 1 min of treatment time. At 1 % concentration and 1 min of treatment time, both TC and TCNE reduced *S.* Enteritidis count in the biofilm by ∼ 2.5 log CFU/ml. Increasing the exposure time to 5 min did not improve the antibiofilm efficacy of 0.5 % TC. However, in the case of 0.5 % TCNE an increase in efficacy with an increase in treatment time was observed. TCNE at 0.5 % dose and 5 min treatment time, reduced *S.* Enteritidis counts significantly by ∼3.5 log CFU/ml as compared to control. At 1 % dose, TC reduced *S.* Enteritidis counts by ∼2.8 log CFU/ml. TCNE 1 % was more effective than TC 1 % and reduced pathogen load by ∼ 3.5 log CFU/ml (*P* < 0.05). At 15 min treatment time, all treatments were effective in reducing pathogen load in the biofilm (*P* < 0.05) as compared to control. TCNE 0.5 % was more effective than TC 0.5 and 1 % and reduced *S.* Enteritidis counts by ∼ 4 log CFU/ml as compared to control.

### Efficacy of TC and TCNE in inactivating mature S. Enteritidis biofilm formed on stainless steel coupons at 25°C

**Fig. 3:**
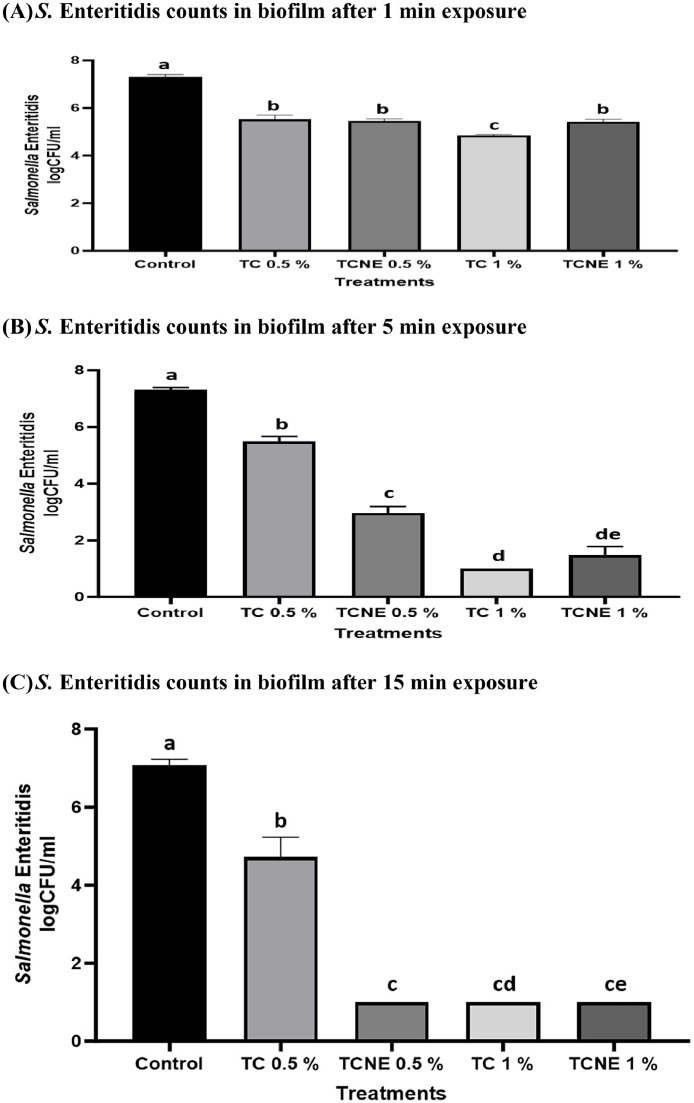
Efficacy of TC and TCNE in Inactivating Mature *S*. Enteritidis Biofilm Formed on Stainless Steel Coupons at 25°C (n = 6).

Fig. 3 shows the efficacy of TC and TCNE in inactivating *S.* Enteritidis biofilm developed on stainless steel surface. In the case of baseline, ∼ 7 log CFU/ml of pathogen load was recovered in the biofilm (data not shown). In case of control, treating the biofilm with DI water for 1, 5, or 15 min did not reduce pathogen load as compared to baseline (Fig. 3A-C). All TC and TCNE treatments were effective in reducing *S*. Enteritidis counts in biofilm by ∼ 1 log CFU, as early as 1 min of treatment time, as compared to control (*P* < 0.05). At 1 min exposure, no difference in the antibiofilm efficacy of 0.5 % TC and 0.5 % TCNE was observed. TC 1 % was slightly more effective than the corresponding TCNE 1 % treatment. After 5 min exposure, 0.5 % TCNE further reduced *S.* Enteritidis counts by an additional 3 log CFU/ml whereas 0.5 % TC did not show an increase in pathogen reduction with increase in exposure time. TC and TCNE, at 1 % dose, significantly reduced *S.* Enteritidis counts by 6.0 log CFU/ml as compared to control. The antibacterial activity of 1 % TCNE was significantly increased with an increase in exposure time from 1 to 5 min on steel coupons. After 15 min of treatment time, 0.5 % TCNE reduced the counts of *S.* Enteritidis by ∼6.5 log CFU/ml. However, 0.5 % TC did not show further reduction in the counts of *S.* Enteritidis as compared to 1- or 5-min exposure. The efficacy of TCNE 0.5 % was found to be similar to 1 % TC or TCNE where a reduction of ∼ 6.5 log CFU/ml in *S.* Enteritidis counts was observed, as compared to control (*P* < 0.05; Fig. 3C).

### Effect of TC and TCNE on S. Enteritidis viability in biofilm

**Fig. 4:**
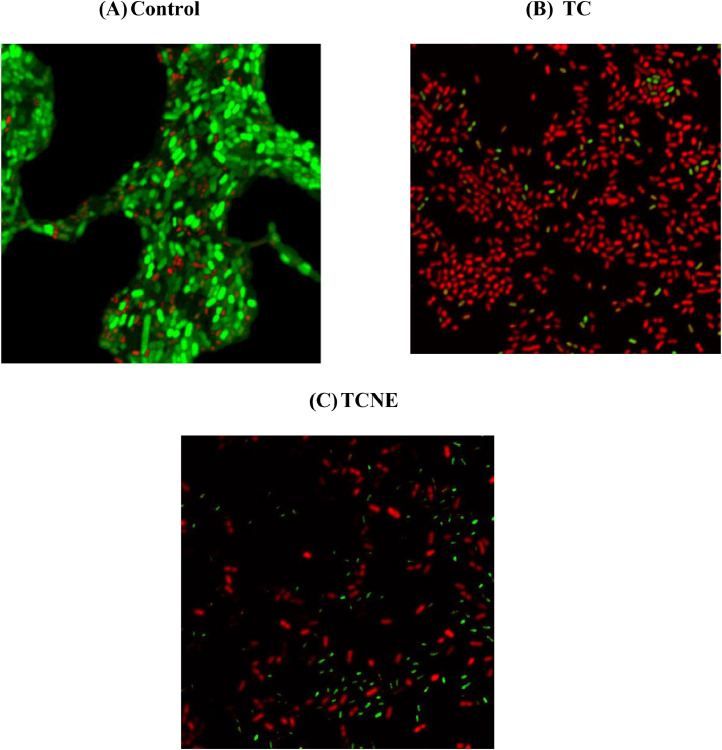
Effect of TC and TCNE on *S*. Enteritidis Viability in Biofilm (63 x magnification).

The effect of TC and TCNE on viability of bacterial cells in the *S*. Enteritidis biofilm was visualized using confocal laser scan microscopy at 63x magnification (Fig. 4). Confocal imaging revealed that the majority of *S.* Enteritidis present in the control biofilm were alive (stained green with SYTO; Fig. 4A), whereas the majority of cells in the biofilm treated with 1 % TC and TCNE for 15 min were dead (stained red with Propidium Iodide; Fig. 4B & C).

### Effect of TC on the expression of S. Enteritidis genes coding for biofilm formation and surface attachment

Table 3 shows the effect of TC on the expression of *S.* Enteritidis genes critical for biofilm formation and surface attachment. TC (0.01 %) significantly modulated the expression of genes encoding for cell surface modifications, motility and stress response. The SIC of TC significantly downregulated surface attachment genes *sipA, sipB, sipC, sopB, hilA,* and *hilC* by more than 3 folds. In addition, gene responsible for motility, *flhD* was also downregulated by ∼ 2.5 folds (*P* < 0.05). Stress response genes, *csgA* and *csgD* were down regulated by ∼ 3 folds as compared to control (*P* < 0.05). The expression of *rpoS* gene (responsible for stress response) was not changed in response to TC (*P* > 0.05).

**Table 3:** Effect of SIC of Trans-cinnamaldehyde on the expression of *S.* Enteritidis genes critical for biofilm formation. Control had a basal level of expression of 1 relative quantification (RQ). *Indicates a significant change in the expression of genes at *P* < 0.05.

Gene	Gene product or function	Relative fold change
*sipA*	Host Cell Invasion (Marcus et al., 2000) and Actin Cytoskeleton Disruption (Yuan et al., 2023)	0.32 ± 0.05*
*sipB*	Host Cell Invasion (Myeni et al., 2013)	0.35 ± 0.03*
*sipC*	Host Cell Invasion (Myeni et al., 2013) and Actin Cytoskeleton Disruption (Yuan et al., 2023)	0.26 ± 0.07*
*sopB*	Host Cell Invasion (Hernandez et al., 2004), manipulation of host cell signaling pathways and the remodeling of the host cell's cytoskeleton (Bakowski et al., 2010)	0.31 ± 0.07*
*hilA*	Activation of Type III Secretion System (T3SS-1) (Ellermeier and Ellermeier, 2005)	0.27 ± 0.08*
*hilC*	Activation of Type III Secretion System (T3SS-1) (Luo et al., 2019)	0.29 ± 0.07*
*flhD*	Flagellar biosynthesis & motility (Li et al., 2017)	0.37 ± 0.04*
*sdiA*	Quorum-sensing regulator (De Almeida et al., 2016)	0.28 ± 0.08*
*csgA*	Curli fiber formation (Barnhart and Chapman, 2006)	0.27 ± 0.04*
*csgD*	Regulation of Curli fiber expression (Gerstel et al., 2006)	0.35 ± 0.04*
*ropS*	Stress Response Regulation (Ibanez-Ruiz et al., 2000)	0.68 ± 0.05

## Discussion

The presence of *S.* Enteritidis biofilms in poultry farm and processing environments poses a significant threat to the safety of poultry products (Yang et al., 2016, Hertwig et al., 2022). Therefore, it is critical to develop effective sanitation strategies for biofilm control. In this study, we selected Trans-cinnamaldehyde oil due to its demonstrated antimicrobial effectiveness against *Salmonella* (Kollanoor-Johny et al., 2008; Jo et al., 2015; Upadhyaya et al., 2015; Upadhyay and Venkitanarayanan, 2016). Several types of emulsifiers, either natural or synthetic, are used for nanoemulsion preparation. We used Tween 80 since it has low cost, low toxicity and high solubilization capacity (Cheng et al., 2017; Sutormin et al., 2022). Moreover, it is widely used as a surfactant in various industrial applications (Cheng et al., 2017; Mehmood and Ahmed, 2020; Das et al., 2022). The stability data (Table 2) suggested that TCNE prepared with Tween 80 are stable for at least 2 months when stored at 4℃. Similar results were observed in our previous publications (Balasubramanian et al., 2022; Allen et al., 2023) and studies from other laboratories (Roldan-Cruz et al., 2016; Wagle et al., 2019b; Udomrati et al., 2020), where nanoemulsions prepared with Tween 80 were found to be stable during storage.

To effectively manage pathogen biofilms, it is crucial to both prevent the formation of new biofilms and eradicate established mature biofilms (Asma et al., 2022; Shrestha, 2022). This two-pronged approach is supported by studies indicating that mature biofilms are more tolerant to antimicrobials (due to protective extracellular polymeric substances) and require a higher dose of chemicals than required for inhibiting biofilm formation (Amalaradjou et al., 2010; Upadhyay et al., 2013; Wagle et al., 2019a; Balasubramanian et al., 2023). Therefore, this study investigated the efficacy of TCNE in both preventing the formation of biofilm and inactivating established biofilm of *S.* Enteritidis. We hypothesized that SIC (compound concentration below the MIC that does not inhibit bacterial growth but modulates their physiology) of TCNE inhibits biofilm formation by modulating the expression of *S.* Enteritidis genes responsible for surface attachment and biofilm development. Additionally, we hypothesized that bactericidal concentration of TCNE rapidly inactivates mature *S.* Enteritidis biofilms developed on polystyrene and stainless-steel surface.

It was observed that SIC of TC and TCNE significantly inhibited *S.* Enteritidis biofilms formation on polystyrene and stainless-steel surface at 48 h (Fig. 1). Similar results were reported with the SIC of TC against *L. monocytogenes* (Upadhyay et al., 2013), where the authors observed significant reductions (∼1.5 log CFU) in *L. monocytogenes* counts in the biofilms developed for 48 h at 25 and 37°C. Similar pattern of reduction was observed by TC against *C. jejuni* (Wagle et al., 2019a), *Streptococcus mutans* (Ngokwe et al., 2024), *Pseudomonas aeruginosa* (Song et al., 2023) and Methicillin-resistant *Staphylococcus aureus* (Kot et al., 2020) biofilms. However, the antibiofilm efficacy of TC and TCNE did not differ significantly on either polystyrene or steel coupons. This may be due to nanoemulsion formulation not significantly enhancing the availability of TC at the biofilm formation site.

Previous studies have shown that SICs of phytochemicals modulate the expression of genes critical for virulence in various pathogenic bacteria (Qiu et al., 2010; Maisuria et al., 2016; Tao et al., 2023), including *L. monocytogenes* (Upadhyay et al., 2012), *C. jejuni* (Upadhyay et al., 2017; Wagle et al., 2019a), and *S.* Enteritidis (Upadhyaya et al., 2013). However, the effect of TC on *S.* Enteritidis genes critical for biofilm formation has not been studied. Therefore, a gene expression study was performed to observe the change in gene expression profile of *S.* Enteritidis in response to sub-inhibitory concentration of TC. The 16S rRNA gene was chosen as the endogenous control due to its stable expression levels between the control and treatment groups. This gene has been utilized as an endogenous control in studies examining the expression of virulence genes in *S.* Enteritidis (Upadhyaya et al., 2013; Yang et al., 2014; Kollanoor Johny et al., 2017). A variety of genes critical for *S.* Enteritidis biofilm formation were selected for this study. For example, SipA, SipB and SipC proteins secreted by *Salmonella* via a type III secretion system (TTSS) encoded by genes such as *sipA, sipB* and *sipC* of the *Salmonella* pathogenicity island 1 (SPI-1) (Lou et al., 2019) those play a crucial role in host cell invasion (Myeni et al., 2013; Marcus et al., 2000), Actin cytoskeleton modulation (Yuan et al., 2023), and biofilm development (Jennings et al., 2012; Lou et al., 2019). The gene *sopB* is responsible for synthesis of outer membrane protein that plays a significant role in Phosphatidylinositol 3-Phosphate formation crucial for bacterial survival and biofilm development (Shafiq et al., 2021). *hilA* is a pivotal regulator of *Salmonella* pathogenicity island 1 (SPI-1), which encodes a type III secretion system (T3SS) crucial for the invasion of host cells. This invasion mechanism is essential for initiating infection and can increase biofilm formation by enhancing bacterial aggregation and adherence to surfaces (Jahan et al., 2022). *hilC*, in conjunction with *hilD* and *rtsA*, constitutes a regulatory network that governs the expression of SPI-1 genes. *hilC* can activate the expression of *hilA*, thereby indirectly affecting the T3SS and biofilm formation. Furthermore, *hilC* has been demonstrated to directly regulate genes involved in biofilm formation, thereby contributing to the stability and persistence of *Salmonella* biofilms (Rana et al., 2021). The *flhD* gene plays a crucial role in the regulation of flagellar biogenesis in *Salmonella*, which is essential for biofilm formation (Li et al., 2017; Albanna et al., 2018; Ma et al., 2022). *sdiA* is a quorum sensing regulator gene in *Salmonella* that influences biofilm development by utilizing autoinducer 1 (AI) molecules produced by other bacteria (Zhang et al., 2022). The *csgA* and *csgD* genes encode structural components of curli fimbriae, which are vital for the formation of the adhesive extracellular matrix of the biofilm (Grantcharova et al., 2010; Liu et al., 2014; El hag et al., 2017; Sokaribo et al., 2020; Harrell et al., 2021). The *rpoS* gene encodes the sigma factor RpoS, which is a central regulator enabling *Salmonella* to adapt to stress conditions and specialized environments. *rpoS* regulates the expression of genes involved in biofilm formation, such as the central regulator *csgD*. It is also reported that specific mutations in the RpoS protein, such as the L193P mutation, can enhance biofilm formation by increasing the expression level and binding activity of *rpoS* to the RNAP and *csgD* gene promoter (Feng et al., 2020; Roy et al., 2021). Results of gene expression analysis revealed that SIC of TC significantly downregulated the expression of *sipA, sipB, sipC, sopB, hilA, hilC, flhD, sdiA, csgA* and *csgD* genes (Table 3). These findings suggest that the antibiofilm effect of TC could potentially be mediated through modulation of genes critical for *S.* Enteritidis surface attachment and biofilm formation. Similar observations have been made by other research groups. For example, Ali et al., 2021 observed that TC attenuated *Enterococcus faecalis* virulence and inhibited biofilm formation. Ying et al., 2019 reported that TC exerted anti virulence effect on *Candida albicans* via farnesol secretion. Kollanoor Johny et al., 2017 reported that *S*. Enteritidis Phase Type (PT) 8 exposed with subinhibitory concentrations of TC at 37°C, significantly down-regulated expression of *S*. Enteritidis PT8 genes involved in flagellar motility, regulation of the *Salmonella* Pathogenicity Island 1, and invasion of intestinal epithelial cells. Upadhyaya et al., 2015 showed that TC downregulated the expression of *S*. Enteritidis virulence genes critical for chicken oviduct colonization. Similar observations have been made against *Pseudomonas aeruginosa* (Song et al., 2023), *Listeria monocytogenes* (Upadhyay et al., 2013), and *Escherichia coli* O157:H7 (Yuan and Yuk, 2019) where sublethal concentration of TC modulated the expression of genes critical for virulence and biofilm formation.

To inactivate mature *S.* Enteritidis biofilms, we used bactericidal concentrations of TC and TCNE. In our study, on majority of treatment dose and time combinations, TC nanoemulsion (TCNE) exhibited greater inactivation efficacy compared to its oil form (Figs. 2A-B; 3A-B). This enhanced efficacy is likely attributable to the more uniform dispersion of TC within the nanoemulsion, which may improve its penetration into biological membranes (Jaiswal et al., 2015). Additionally, nanoemulsions have a higher surface area-to-volume ratio compared to bulk oils, facilitating more effective interactions with target sites and leading to increased biological activity (Gupta et al., 2016). Similar observations have been made in previous studies where eugenol nanoemulsion and TC nanoemulsions were more effective in inactivating foodborne pathogens on eggs, cantaloupes and in biofilms than their corresponding oil forms (Balasubramanian 2023, 2022; Allen et al., 2023).

To validate the inactivation results, we visualized the treated biofilms using bacterial viability assay. In the control samples, the predominant *S.* Enteritidis cells were live (green), whereas the majority of *S.* Enteritidis cells were dead (red) after treatment (Fig. 4A-C). The green fluorescence for live bacteria and red fluorescence for dead bacteria can be attributed to the use of SYTO 9 and propidium iodide (PI) stains. SYTO 9 is cell-permeable and can enter all bacterial cells, staining them green. In contrast, PI is membrane-impermeable and can only enter cells with damaged membranes, staining them red. This indicates that TCNE has killed *S.* Enteritidis by disrupting the cell membrane, allowing PI to enter the cells. Similar results of confocal microscopy were reported previously with TC, EG and CR against *L. monocytogenes* (Upadhyay et al., 2013) and *C. jejuni* (Wagle et al., 2019a) biofilms.

In conclusion, TCNE has shown better efficacy in inactivating mature *S.* Enteritidis biofilms on polystyrene and stainless-steel surface at 25°C as compared to TC oil alone. This reduction could potentially lead to reduced product contamination in the processing plant. However, a correlation between a reduction in *S.* Enteritidis biofilm counts and corresponding reductions in pathogen load on carcass has not been conducted and could be a focus of future research. In addition, the impact of inherent surface microflora on *S.* Enteritidis biofilm and phytochemical nanoemulsion efficacy should be evaluated in future studies. Overall, nanoemulsion of TC has the potential to be used as a natural, safe and effective formulation to control S. Enteritidis biofilm in farm environment.

## Declaration of competing interest

**The authors declare no conflicts of interest** related to the manuscript entitled “*Trans*-cinnamaldehyde nanoemulsion reduces *Salmonella* Enteritidis biofilm on steel and plastic surfaces and downregulates expression of biofilm associated genes*.*” that is bring submitted for exclusive consideration of publication as an original research paper in Poultry Science.
